# Body Composition, Fitness, and Mental Health in Preadolescent Children

**DOI:** 10.1001/jamanetworkopen.2025.28868

**Published:** 2025-08-26

**Authors:** Bianca Braun, Naiman A. Khan, Charles H. Hillman, Lauren B. Raine

**Affiliations:** 1 Institute for Cognitive and Brain Health, Interdisciplinary Science and Engineering Complex, Northeastern University, Boston, Massachusetts; 2 Department of Physical Therapy, Movement and Rehabilitation Sciences, Bouvé College of Health Sciences, Northeastern University, Boston, Massachusetts; 3 Department of Medical Sciences, Bouvé College of Health Sciences, Northeastern University, Boston, Massachusetts; 4 Department of Psychology, Northeastern University, Boston, Massachusetts; 5 Department of Health and Kinesiology, University of Illinois at Urbana-Champaign; 6 Division of Nutritional Sciences, University of Illinois at Urbana-Champaign; 7 Neuroscience Program, University of Illinois at Urbana-Champaign; 8 Personalized Nutrition Initiative, University of Illinois at Urbana-Champaign; 9 Beckman Institute for Advanced Science and Technology, University of Illinois at Urbana-Champaign

## Abstract

**Importance:**

There is an increasing prevalence of poor fitness, concurrent with rising obesity, anxiety, and depression among children in the US. However, the association of fitness, adiposity, and lean tissue with anxiety and depression in preadolescent children is understudied.

**Objective:**

To estimate differences in the association between specific tissues (adipose and lean tissue) and cardiorespiratory fitness with anxiety and depression symptoms among children.

**Design, Setting, and Participants:**

Cross-sectional study between 2019 and 2023. Participants were a volunteer sample of children aged 8 to 11 years from the Greater Boston, Massachusetts, region without prior mental health diagnosis.

**Exposures:**

Body composition assessed via dual-energy x-ray absorptiometry (body fat percentage [BF%], visceral adipose tissue [VAT], and lean mass) and cardiorespiratory fitness measured by peak oxygen uptake (VO_2_peak) relative to body weight.

**Main Outcomes and Measures:**

Children self-reported trait anxiety (State-Trait Anxiety Inventory for Children) and depressive symptoms (Children’s Depression Inventory). Independent multivariable hierarchical regression analyses were conducted for each outcome with sociodemographic factors in step 1 and BF%, VAT, lean mass, or fitness in step 2.

**Results:**

Among 207 children (mean [SD] age, 10.0 [0.7] years; 119 male [57.5%]; 1 [0.5%] American Indian or Alaska Native, 26 [12.6%] Asian, 39 [18.8%] Black or African American, 120 [58%] White, and 21 [10.1%] mixed race or other), anxiety was positively associated with BF% (standardized regression coefficient [β] = 0.15; 95% CI, 0.23 to 6.33; *P* = .04) and VAT (β = 0.15; 95% CI, 0.04 to 1.82; *P* = .04), and negatively associated with lean mass (β = −0.16; 95% CI, −14.77 to −1.21; *P* = .02) and VO_2_peak (β = −0.19; 95% CI, −0.28 to −0.04; *P* = .01). Depression showed positive associations with VAT (β = 0.27; 95% CI, 0.34 to 1.11; *P* < .001), and negative associations with lean mass (β = −0.16; 95% CI, −6.39 to −0.43; *P* = .03) and VO_2_peak (β = −0.16; 95% CI, −0.11 to −0.01; *P* = .03).

**Conclusions and Relevance:**

In this cross-sectional study of preadolescent children, greater lean mass and higher fitness were associated with fewer anxiety and depression symptoms, while higher VAT was associated with increased symptoms of both. BF% was only associated with greater anxiety. These findings highlight the roles of body composition in mental health and underscore the value of early identification of physical health markers to support children’s well-being and development.

## Key Points

**Question:**

How are body composition and cardiorespiratory fitness associated with mental health in preadolescent children?

**Findings:**

In this cross-sectional study of 207 preadolescent children, adiposity measured via dual-energy x-ray absorptiometry was associated with higher anxiety and depression symptoms. Higher levels of lean mass and cardiorespiratory fitness were associated with fewer anxiety and depression symptoms.

**Meaning:**

These findings suggest that excess adiposity may be a factor associated with risk for poorer mental health in preadolescent children whereas higher lean mass and fitness may be protective, highlighting the importance of assessing these factors for targeted interventions and improving children’s developmental trajectory.

## Teaser

This cross-sectional study evaluates differences in the associations of adipose and lean tissues and cardiorespiratory fitness with anxiety and depression symptoms among children in the Boston area.

## Introduction

Worldwide, nearly 20% of children and adolescents experience mental health challenges.^1,2^ The most common mental health challenges facing children are anxiety and depression,^3^ which grew by nearly 30% between 2016 and 2020. These rates do not account for subclinical symptoms that are increasingly widespread risk factors for future development of clinically diagnosed anxiety and depression disorders.^2^ Approximately 40% of adolescents currently experience persistent feelings of sadness or hopelessness,^4^ which are a defining feature of depression.^5,6^ Anxiety in children is characterized by fears and worries that interfere with school, home, or play, and extend beyond the typical fears and worries present in children.^5^ These mental health challenges are important to consider because they affect the developmental trajectory of children and can have long-term adverse effects,^7^ with more than half of all adult mental health challenges beginning in childhood or adolescence.^7,8^ In children, depression negatively impacts academic and cognitive performance,^9^ while over time, the physiological effects of chronic anxiety and depression can lead to heart disease.^10^ The consequences of anxiety and depression are long-lasting and can be debilitating. Hence, early investigations into preadolescent health factors that may influence the development of anxiety and depression are crucial.

Health factors related to childhood mental health may include body composition and cardiorespiratory fitness (hereafter, fitness). Unfortunately, most children do not meet the 2018 Physical Activity Guidelines for Americans,^11^ with nearly 60% of children in the US lacking healthy fitness levels.^12^ A recent review found that higher fitness in children and adolescents was related to better health-related quality of life, including physical and psychological well-being, quality of peer relationships, and school functioning.^13^ Few studies have investigated the relationship between fitness and mental health, specifically anxiety and depression, in children. Higher fitness has been associated with fewer depressive symptoms and greater feelings of self-worth,^14^ as well as with positive mental health outcomes and reduced risks of anxiety and depression.^15^ Overall, research on the relationship between fitness and anxiety and depression in children under 10 is minimal and poorly understood, urging investigation using sensitive, best-practice measures of fitness.

Concurrent with recent decreases in fitness, rates of obesity (body mass index [BMI] ≥95th percentile)^16^ among youth have increased to approximately 20%.^17^ Two international studies^18,19^ have identified increased BMI and obesity as critical risk factors for anxiety and depression in youth. Equivalent studies have not been conducted in the US. However, while BMI is the most frequently used anthropometric method in the literature for assessing obesity^20^ and a useful screening tool to estimate disease risk, it is an indirect measure and does not assess body composition.^21^ More precise body composition estimates can be achieved via dual-energy x-ray absorptiometry (DXA), which assesses total adipose tissue, visceral adipose tissue (VAT), subcutaneous abdominal adipose tissue (SAAT), and lean mass.^22^ Distinguishing these tissue types is important because while excess VAT has been associated with inflammation, metabolic syndrome, cardiovascular disease, and several malignant neoplasms,^23,24^ health implications of SAAT are inconclusive.^23,25,26^ The mental health implications of lean mass are also understudied in children. While lower lean mass in older adults has been associated with greater depression severity,^27^ findings are mixed,^28^ and limited research has examined these associations in youth. One study^29^ reported that children and middle-aged adults with moderate to severe depression had significantly lower lean mass and higher total and regional fat mass. Body fat percentage (BF%) was also positively associated with anxiety and depression in another study of female adolescents, with no associations between adipose tissue location and anxiety or depression.^30^ Prior research suggests that excess adiposity may be a risk factor for adverse mental health outcomes in adolescents. However, most studies have relied on weight status, as measured using BMI-for-age and -sex, with only 2 studies^29,30^ investigating BF%. The differential relationships between BF%, VAT, and lean mass with mental health have not been investigated in preadolescent children to our knowledge, despite the potential negative health consequences of these body composition measures.

Together, evidence suggests adolescents with excess adiposity may be at increased risk for mental health challenges.^18,31^ Emerging evidence in youth also proposes that fitness is protective against mental health challenges, with higher fit individuals experiencing lower anxiety and depression.^15^ Despite childhood being a critical period for physical and mental development, few studies have investigated the cause of and physical health factors contributing to mental health concerns. Specifically, we know of no evidence investigating the differential role of adipose vs lean tissue and fitness on mental health in preadolescents. Investigating these associations will enable the development of targeted public health strategies and interventions that promote both physical and mental health early on. It could also inform policies that integrate physical activity and nutrition into school and community programs, fostering overall well-being in youth.

The present study is the first we know of to investigate the differential role of adiposity, lean tissue, and fitness with anxiety and depression symptoms in a large sample of preadolescent children. It is also the first we know of to use DXA-derived body composition measures and a VO_2_peak test, the criterion standard for cardiorespiratory fitness assessment.^32^ We hypothesized that a higher amount of BF% and adipose tissue would be associated with greater symptoms of anxiety and depression, whereas higher lean mass and fitness would be associated with fewer anxiety and depression symptoms.

## Methods

### Participants

This cross-sectional study included data from preadolescent children^33^ who participated in research studies at the Center for Cognitive and Brain Health at Northeastern University in Boston, Massachusetts between 2019 and 2023. A detailed description of the procedures has been previously published.^34^ All assessments took place on a single day. Eligibility requirements included: (1) being capable of exercise per the physical activity readiness questionnaire for children,^35^ (2) IQ of 85 or higher, (3) no diagnosis of cognitive or physical disability, (4) no neuropsychological medication, (5) normal or corrected-to-normal vision, and (6) fluent in English. Nineteen participants with missing data (3 participants missing VAT, 6 missing mother’s education, and 10 missing pubertal development) were excluded from the analysis. Participant demographics (age, sex, race, pubertal development, and mother’s education) did not differ significantly between included and excluded participants (eTable 1 in

Supplement 1). All children provided written assent, and legal guardians provided written informed consent in accordance with the Northeastern University institutional review board. Guardians completed questionnaires reporting their child’s age, sex, race, pubertal development (via a modified Tanner staging questionnaire),^36^ and mother’s education (as a proxy of socioeconomic status). Race was parent-reported using the following categories: American Indian or Alaska Native, Asian, Black or African American, White, mixed race or other. Race was assessed due to previously documented racial differences in anxiety and depression symptoms among children.^37^ This study followed the Strengthening the Reporting of Observational Studies in Epidemiology (STROBE) reporting guideline for cross-sectional studies.

### Measures

#### Body Composition

Standing height and weight measurements were completed using a Health o Meter Professional 500KL digital patient weighing scale with stadiometer. BMI was calculated as weight in kilograms divided by height in meters squared, and BMI percentile was calculated based on CDC growth charts.^38^ Participants’ body composition was estimated using DXA (Lunar iDXA, version 18; GE Healthcare), an accurate, reliable, and highly precise method for children.^39^ Body composition measures included estimates of VAT (cm^3^), total body less head (TBLH) lean mass (grams), TBLH fat mass (grams), and TBLH total mass (grams). To adjust for child size, BF% was calculated as TBLH fat mass/TBLH total mass and lean mass as TBLH lean mass/TBLH total mass.

#### Cardiorespiratory Fitness

Children completed a peak oxygen uptake test (treadmill: Trackmaster TMX428; metabolic cart: Quark CPET, OMNIA) using a modified Balke protocol. Relative VO_2_peak (VO_2_peak; mL/kg/min, 20-second averaging) indicated maximal effort by: (1) plateau in VO_2_ with less than a 2 mL/kg/min increase despite rising grade, (2) peak heart rate of 185 beats/minute or higher and heart rate plateau,^40^ (3) respiratory exchange rate^41^ of 1.0 or higher, or (4) rate of perceived exertion^42^ of 8 or higher.

#### Mental Health

##### Depression

Symptoms were assessed via child self-report using the 12-item short form of the Child Depression Inventory (CDI), 2nd edition,^43^ a valid and reliable screening tool for those aged 7 to 17 years.^44^ Scores range from 0 to 24, with higher scores indicating greater severity. The CDI shows strong convergent validity with structured clinical interviews and other depression scales.^45^

##### Anxiety

Stable anxiety tendencies were assessed using the trait subscale of the State-Trait Anxiety Inventory for Children (STAIC-T),^46^ a 20-item validated and reliable self-report measure completed by children aged 8 to 14 years.^47,48^ Scores range from 20 to 60, with higher scores indicating greater severity. The STAIC-T demonstrates convergent validity and discriminates well between youth with and without anxiety disorders.^49^

### Statistical Analysis

Body composition measures (BMI, BF%, VAT, and lean mass) were log-transformed to satisfy model assumptions and improve model fit. Analysis of log-transformed variables identified 4 outliers in VAT, 1 in lean mass, 1 in VO_2_peak, 1 in anxiety, and 2 in depression. Each value only marginally exceeded 3 SDs and was verified as plausible and accurate. Consequently, these data points were included. To create more evenly distributed groups, race and mother’s education were dichotomized into White and other groups and less than advanced degree and advanced degree or more, respectively. Associations between STAIC-T and CDI with demographic variables were determined via Pearson product-moment correlation (for age) and 4 independent 1-way analyses of variance (ANOVA; for sex, race, pubertal development, and mother’s education).

Next, separate multiple multivariable hierarchical linear regressions were conducted for each dependent measure. Any demographic variables that were significantly associated with the dependent measure of interest were included in step 1. Sex was included in step 1 for all models due to the existing literature on sex differences in body composition and fitness.^50,51^ In a separate step 2, body composition (BMI, BF%, VAT, and lean mass) or fitness (VO_2_peak) was included for each dependent variable (STAIC-T or CDI). Multiple comparisons for models were controlled using Holm-Bonferroni^52^ with 5 comparisons (BMI, BF%, VAT, lean mass, and VO_2_peak) for each outcome variable (all adjustments significant between *P* < .01 and *P* < .05) (eTable 2 in Supplement 1). All models and analyses were performed with a 2-tailed α level  = .05 using R Statistical Software version 4.1.1 (R Project for Statistical Computing).

## Results

The final sample consisted of 207 preadolescent children (mean [SD] age, 10.0 [0.7] years; 119 [57.5%] male; 1 [0.5%] American Indian or Alaska Native, 26 [12.6%] Asian, 39 [18.8%] Black or African American, 120 [58%] White, and 21 [10.1%] mixed race or other). Demographic variables, body composition, fitness, and symptoms of anxiety and depression can be found in Table 1.

**Table 1.** Participant Characteristics

Characteristic	Participants (N = 207), No. (%)^a^
Age, mean (SD) [range], y	10 (0.7) [8.1-11.5]
Sex	
Female	88 (42.5)
Male	119 (57.5)
Race^b^	
American Indian or Alaska Native	1 (0.5)
Asian	26 (12.6)
Black or African American	39 (18.8)
White	120 (58)
Mixed race or other	21 (10.1)
Mother’s education (proxy for socioeconomic status)	
Less than advanced degree	94 (45.4)
Advanced degree or more	113 (54.6)
Pubertal development	
Tanner stage I	147 (71)
Tanner stage II	51 (24.6)
Tanner ≥stage III	9 (4.3)
Body composition measures	
BMI, mean (SD) [range]^c^	18.8 (4.0) [12.5-35]
BMI percentile, mean (SD) [range], %	60.4 (30.5) [0.1-99.4]
% Body fat, mean (SD) [range], %	30 (8.5) [12.6-53.9]
Visceral adipose tissue, mean (SD) [range], cm^3^^d^	145 (160) [2-1075]
Adjusted total body less head lean mass, mean (SD) [range], g	0.67 (0.08) [0.44-0.83]
VO_2_peak, mean (SD) [range], mL/kg/min	41.5 (7.55) [18.2-62.4]
Mental health	
Trait anxiety, mean (SD) [range]	32.6 (6.37) [20-54]
Depression, mean (SD) [range]	3.2 (2.82) [0-14]

Abbreviations: BMI, body mass index; VO_2_peak, peak oxygen consumption.

^a^ Percentages have been rounded and may not total 100.

^b^ Exact and only categories provided to participants to self-select from with no breakdown of mixed race or other collected.

^c^ BMI calculated as weight in kilograms divided by height in meters squared.

^d^ VAT estimated using young adult parameters.

### Correlations

No significant correlations were found between age and trait anxiety (*r*_205_ = −0.02; 95% CI, −0.15 to 0.12; *P* = .81) or depression (*r*_205_ = 0.03; 95% CI, −0.11 to 0.16; *P* = .69). Correlations between categorical demographic variables and mental health outcomes are presented in ANOVA Table 2. Only pubertal development correlated with mental health; sex, race, and mother’s education were not correlated with STAIC-T or CDI scores.

**Table 2.** Analysis of Variance Between Participant Demographics and Mental Health Variables^a^

Outcome and covariate	*df*	Sum of squares	Mean squares	*F* Statistic	*P* value
STAIC-T					
Sex	1	6.813	6.813	0.167	.68
Residuals	205	8346.491	40.715	NA	NA
Race	1	57.910	57.910	1.431	.23
Residuals	205	8295.394	40.465	NA	NA
Tanner	1	216.000	216.000	5.442	.02^b^
Residuals	205	8137.304	39.694	NA	NA
Mother’s education	1	94.898	94.898	2.356	.13
Residuals	205	8258.406	40.285	NA	NA
CDI					
Sex	1	0.033	0.033	0.004	.95
Residuals	205	1638.614	7.993	NA	NA
Race	1	10.044	10.044	1.264	.26
Residuals	205	1628.603	7.944	NA	NA
Tanner	1	72.114	72.114	9.437	.002^c^
Residuals	205	1566.533	7.642	NA	NA
Mother’s education	1	11.243	11.243	1.416	.24
Residuals	205	1627.404	7.939	NA	NA

Abbreviations: CDI, Child Depression Inventory; NA, not applicable; STAIC-T, State Trait Anxiety Inventory for Children–Trait Anxiety.

^a^ Findings are shown for the two 4-way analyses of variance that were performed with demographics (sex, race, Tanner pubertal development, mother’s education) for both outcome variables independently: trait anxiety and depression.

^b^ Denotes statistical significance of *P* < .05.

^c^ Denotes statistical significance of *P* < .01.

### Hierarchical Regression Models

All significant findings reported in subsequent sections remain significant after adjusting for multiple model comparisons using Holm-Bonferroni (eTable 2 in Supplement 1). Step 1 for STAIC-T and CDI included sex and pubertal development. BMI was not significantly associated with anxiety (β = 0.14; 95% CI, −0.17 to 9.02; *P* = .06) or depression (β = 0.14; 95% CI, −0.01 to 4.03; *P* = .05). These findings, along with the complete regression outputs, are included in eTables 3 and 4 in Supplement 1.

#### Trait Anxiety

**Figure 1.**
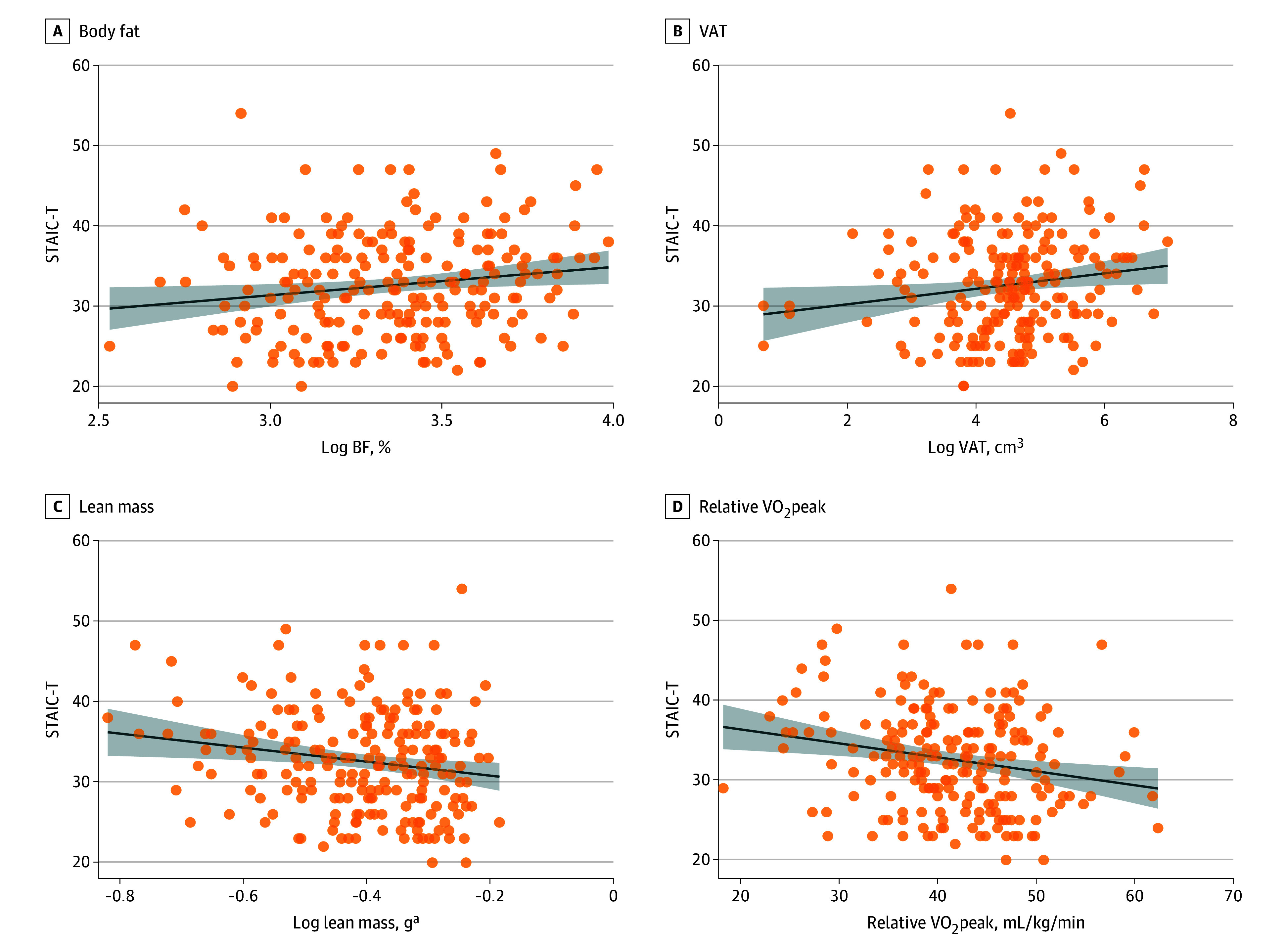
Body Composition and Fitness Associations With Trait Anxiety Graphs show individual data points (circles) and best-fitted regression lines with 95% CIs (shaded areas), without covariate adjustment. BF indicates body fat; STAIC-T, State Trait Anxiety Inventory for Children–Trait Anxiety; VO_2_peak, peak oxygen uptake; VAT, visceral adipose tissue. ^a^Lean mass refers to the adjusted total body less head lean mass which was calculated in relation to the child’s size.

In step 2, associations were found for body composition and fitness measures (Figure 1; eTable 3 in Supplement 1). Higher BF% was associated with higher STAIC-T (β = 0.15; 95% CI, 0.23 to 6.33; *P* = .04). Higher VAT was associated with higher STAIC-T (β = 0.15; 95% CI, 0.04 to 1.82; *P* = .04). Higher lean mass was associated with lower STAIC-T (β = −0.16; 95% CI, −14.77 to −1.21; *P* = .02). Significant associations were also found for VO_2_peak, with higher VO_2_peak associated with lower STAIC-T (β = −0.19; 95% CI, −0.28 to −0.04; *P* = .01).

#### Depression

**Figure 2.**
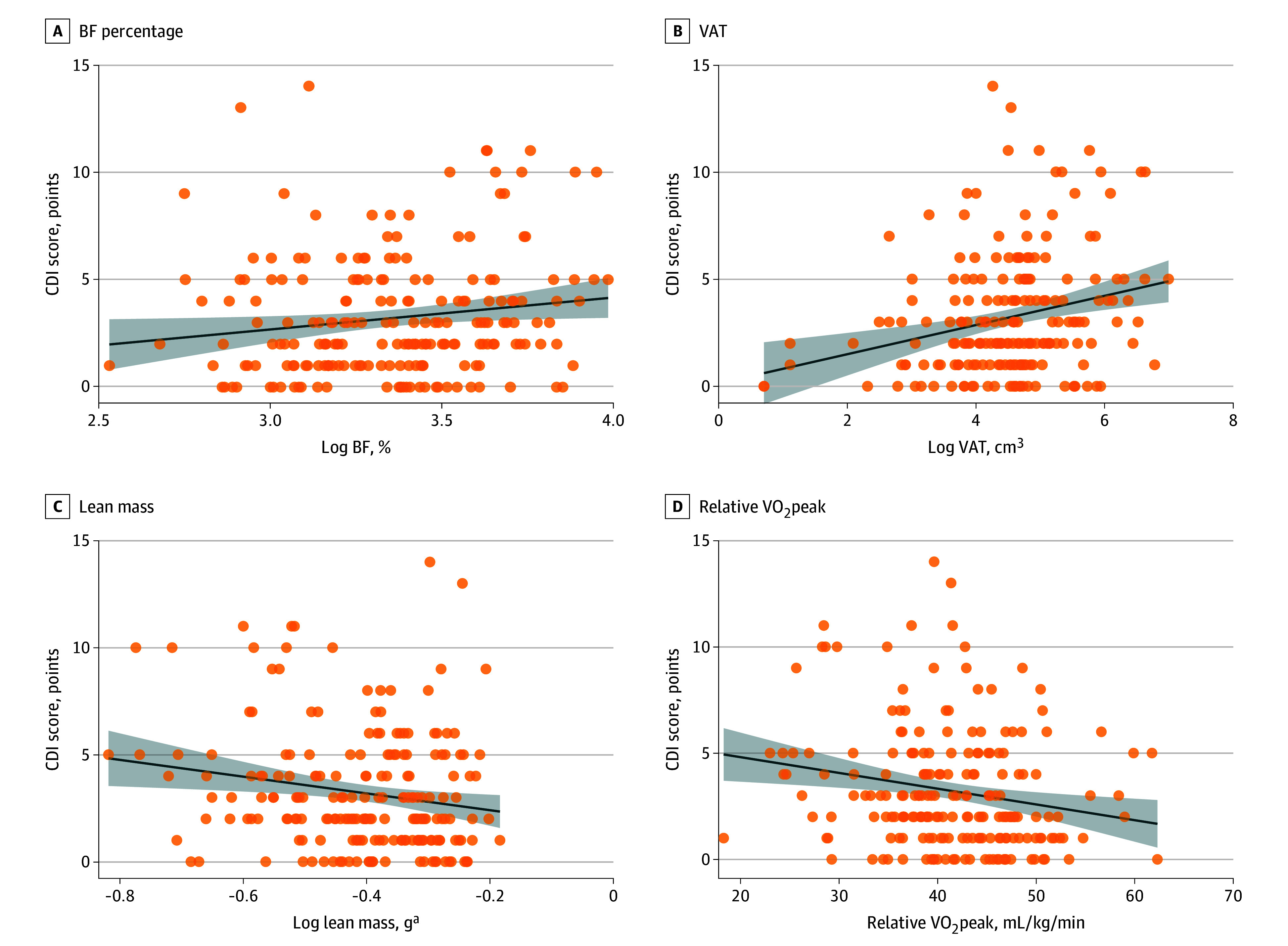
Body Composition and Fitness Associations With Depression Graphs show individual data points (circles) and best-fitted regression lines with 95% CIs (shaded areas), without covariate adjustment. BF indicates body fat; CDI, Child Depression Inventory; VO_2_peak, peak oxygen uptake; VAT, visceral adipose tissue. ^a^Lean mass refers to the adjusted total body less head lean mass, which was calculated in relation to the child’s size.

In step 2, associations were found for body composition and fitness measures except for BF% (Figure 2; eTable 4 in Supplement 1). BF% was not associated with CDI (β = 0.13; 95% CI, −0.08 to 2.61; *P* = .07). However, higher VAT was significantly associated with higher CDI (β = 0.27; 95% CI, 0.34 to 1.11; *P* < .001) and higher lean mass was associated with lower CDI (β = −0.16; 95% CI, −6.39 to −0.43; *P* = .03). Lastly, significant associations were found for VO_2_peak, with higher VO_2_peak associated with lower CDI (β = −0.16; 95 % CI, −0.11 to −0.01; *P* = .03).

## Discussion

The purpose of this investigation was to understand the association between sensitive measures of body composition, fitness, and mental health in preadolescent children. In a well-characterized cohort of 207 children, body composition (BF%, VAT, and lean mass) and fitness were significantly but distinctly associated with anxiety and depression. Lean mass and VO_2_peak were negatively associated with both outcomes; children with greater lean mass and higher fitness levels reported fewer symptoms of anxiety and depression. In contrast, higher BF% and VAT were associated with greater anxiety symptoms, with VAT also associated with increased depressive symptoms. Notably, BF% showed no association with depression, and BMI was unrelated to either outcome, although both trended in the expected direction and aligned with prior research. By using sensitive measures of body composition rather than conventional anthropometric methods, these findings underscore the importance of distinguishing between tissue types when examining mental health concerns in children.

The present study suggests that the association between body composition and mental health is complex and highlights the potential significance of adipose tissue location, such as VAT. One possible mechanism is the abnormal secretion of proinflammatory adipokines and lipokines, which increase with VAT,^53^ and have been implicated in heightened neuroinflammatory and dysregulation of mood-related brain regions, including the hippocampus and amygdala.^54^ These processes may underpin the bidirectional link between obesity and depression.^54^ While a prior study^30^ in female adolescents reported no associations between adipose tissue location and mental health symptoms, our findings suggest that such associations may emerge earlier in development, before pubertal shifts in fat distribution, hormones, or neurodevelopmental processes mask detectability—even with sensitive tools. This may reflect a developmentally sensitive window in which VAT is associated with affective symptoms.

In contrast, lean mass and cardiorespiratory fitness may act as protective factors. Fitness, enhanced through regular physical activity, has been linked to reduced stress reactivity and more adaptive neuroendocrine and physiological responses to physical and psychosocial stressors.^55^ Physical activity may promote an anti-inflammatory state, improve immune function, and enhance neuroplasticity and growth factor expression.^55^ Although less studied, lean mass acts as an endocrine organ, with skeletal muscle releasing myokines (eg, Irisin, BDNF, and IL-6) that can modulate inflammation and impact brain health.^56^ These biological adaptations can build resilience by blunting stress responses over time, increasing emotional stability and buffering against anxiety and depression.^55^ This framework—referred to as the stress-adaptation hypothesis^57^—is supported by our findings, as children with greater fitness and lean mass exhibited fewer anxiety and depression symptoms.

Although the underlying mechanisms remain underexplored in pediatric samples, studies in adolescents and adults suggest consistent patterns. A meta-analysis in adults found that low and medium fitness levels were associated with a 47% and 23% increased risk of anxiety or depression, respectively, compared with high fitness, suggesting a dose-response association.^58^ Two studies in children^15,58^ found that higher fitness was associated with lower risks of anxiety and depression and that higher fitness protected against depression over time. Moreover, individuals diagnosed with depression tend to exhibit lower muscle mass and higher fat mass, an association observed across adolescents and adults.^59^ A similar association between lower muscle mass and greater anxiety has been shown in 2 studies of middle-aged adults.^60,61^ Our findings extend this, suggesting these associations may originate in childhood, emphasizing the importance of early prevention strategies targeting fitness and lean mass development.

Psychosocial and behavioral factors may also influence these associations. Lower fitness or higher adiposity may contribute to poor body image, increased screen time, and social withdrawal, which have all been linked to mental health concerns.^62,63,64,65^ Similarly, peer-related experiences, such as bullying, weight-based teasing, or participating in physical activity with peers, may shape the emotional impact of fitness and body composition differently by sex. These pathways warrant further future exploration, as they may influence observed associations in real-world settings.

### Strengths and Limitations

The current study possesses several strengths: (1) sensitive DXA-derived body composition measures, (2) a novel investigation of lean tissue mass, (3) assessment of fitness using the criterion standard VO_2_peak, and (4) valid and reliable self-reported mental health symptoms from (5) understudied preadolescent participants. Previous research has largely focused on BMI, which fails to differentiate between tissue types. Our findings address a critical gap in the literature and suggest that more precise measures may offer clearer insights into childhood mental health.

The present study should be interpreted with several limitations. As a cross-sectional study, causality cannot be inferred. Future longitudinal studies examining within-person variation in mental health, body composition, and fitness are needed to clarify developmental trajectories, mechanisms, and bidirectional relationships. Additionally, relevant factors, such as nutrition, physical activity (including type and peer dynamics), sleep, body image, and social experiences like weight-based teasing and social media use, were not measured but may influence these relationships. Although absent in our dataset, future research should consider these sociocultural dimensions. Our volunteer sample of generally healthy, unmedicated children introduces potential self-selection bias and limits generalizability, particularly to clinical populations and those with limited access to care. Future research should investigate these associations in clinical, socioeconomically diverse, and more representative samples. Although Tanner staging was included as a covariate, it may overestimate pubertal status in children with higher adiposity.^66,67^ Furthermore, while VO_2_peak is the criterion standard for assessing cardiorespiratory fitness, the validity of secondary criteria to confirm maximal effort in children has been questioned,^68^ potentially adding variability in fitness estimates. Finally, while statistically significant, effect sizes were relatively small. These findings should be viewed as preliminary indicators of associations that, with replication and further validation, may inform early identification efforts and guide preventative interventions.

## Conclusions

This cross-sectional study of children aged 8 to 11 years provides insight into the differential associations between tissue types and mental health symptoms in children. Our findings suggest that fitness and lean mass may serve as protective factors, while adiposity may confer risk. These distinctions, detectable even in preadolescence, could support early identification of children at greater risk for mental health symptoms. Integrating modifiable factors like fitness and body composition into routine pediatric assessments may help guide preventative care and early intervention to improve children’s mental health outcomes.

## Notes

Supplement 1. **eTable 1. **Observed Characteristics Between Participants With Complete and Incomplete Data **eTable 2. **Holm-Bonferroni Multiple Comparisons Adjustment for Trait Anxiety and Depression Models **eTable 3. **Hierarchical Regression Table for Trait Anxiety Outcomes of Complete Data Sample **eTable 4.** Hierarchical Regression Table for Depression Outcomes of Complete Data Sample

Supplement 2.
Data Sharing Statement
