# Supplementary material for: Body Composition, Fitness, and Mental Health in Preadolescent Children
Source: JAMA Netw Open. 2025 Aug 26;8(8):e2528868. doi: 10.1001/jamanetworkopen.2025.28868 (PMC12381672; doi:10.1001/jamanetworkopen.2025.28868)
Supplement: Supplement 1. — eTable 1. Observed Characteristics Between Participants With Complete and Incomplete Data eTable 2. Holm-Bonferroni Multiple Comparisons Adjustment for Trait Anxiety and Depression Models eTable 3. Hierarchical Regression Table for Trait Anxiety Outcomes of Complete Data Sample eTable 4. Hierarchical Regression Table for Depression Outcomes of Complete Data Sample [file jamanetwopen-e2528868-s001.pdf]

Supplemental Online Content

Braun B, Khan NA, Hillman CH, Raine LB. Body composition, fitness associations, and mental health in preadolescent children. *JAMA Netw Open*. 2025;8(8):e2528868.
doi:10.1001/jamanetworkopen.2025.28868

eTable 4. Observed Characteristics Between Participants With Complete and Incomplete Data

eTable 1. Holm-Bonferroni Multiple Comparisons Adjustment for Trait Anxiety and Depression Models

eTable 2. Hierarchical Regression Table for Trait Anxiety Outcomes of Complete Data Sample

eTable 3. Hierarchical Regression Table for Depression Outcomes of Complete Data Sample

This supplemental material has been provided by the authors to give readers additional information about their work.

© 2025 Braun B et al. *JAMA Netw Open*.

eTable 1. Observed Characteristics Between Participants With Complete and Incomplete Data[a,]

Characteristic	Participants, No. (%)[b] Complete sample (n=207)	Incomplete sample (n=19)	P Value
**Sex**	n=207	n=19	0.50[c]
**Female**	88 (42.5)	6 (31.6)	
**Male**	119 (57.5)	13 (68.4)	
**Race[f]**	n=207	n=14	0.43[d]
**American Indian or Alaska Native**	1 (0.5)	0	
**Asian**	26 (12.6)	1 (7.1)	
**Black or African American**	39 (18.8)	1 (7.1)	
**White or Caucasian**	120 (58)	9 (64.3)	
**Mixed or Other, No. (%)**	21 (10.1)	3 (21.4)	
**Mother's education (proxy for socioeconomic status)**	n=207	n=13	0.15[d]
**Less than advanced degree, No. (%)**	94 (45.4)	9 (69.3)	
**Advanced degree or more, No. (%)**	113 (54.6)	4 (30.8)	
**Age, mean (SD), y**	n=207<br>10 (0.7)	n=19<br>10 (1)	0.30[e]
**Pubertal development**	n=207	n=3	0.60[d]
**Tanner = Stage I**	147 (71%)	3 (100%)	
**Tanner = Stage II**	51 (24.6%)	0	
**Tanner ≥ Stage III**	9 (4.3%)	0	
**Body composition measures**			
**BMI, mean (SD)**	n=207<br>18.8 (4.0)	n=19<br>17.2 (2.4)	0.17[e]
**BMI percentile, mean (SD), %**	n=207<br>60.4 (30.5)	n=19<br>50.7 (33.0)	014[e]
**Percent body fat, mean (SD), %**	n=207<br>30 (8.5)	n=19<br>24.5 (5.4)	0.005[e]
**Visceral adipose tissue, mean (SD), cm^3**	n=207<br>145 (160)	n=16<br>94.88 (67.8)	0.31[e]
**Adjusted total body less head lean mass, mean (SD), g**	n=207<br>0.67 (0.08)	n=19<br>0.72 (0.05)	0.005[e]
**Relative VO_2peak, mean (SD), mL/kg/min**	n=207<br>41.5 (7.55)	n=19<br>45.5 (6.45)	0.03[e]
**Mental health**			
**Trait anxiety (STAIC-T)**	n=207<br>32.6 (6.37)	n=19<br>32.4 (6.24)	0.19[e]
**Depression (CDI)**	n=207<br>3.2 (2.82)	n=19<br>3.16 (2.79)	0.38[e]

© 2025 Braun B et al. *JAMA Netw Open*.

[a] The table presents a comparison of the observed characteristics between the sample of those with incomplete and with complete data included in the final analysis.

[b] Percentages have been rounded and may not total 100.

[c] Chi-square test was conducted for categorical variables.

[d] Fisher's exact test was conducted for categorical variables if cell counts <5.

[e] Wilcoxon rank-sum test was conducted for continuous variables.

[f] Exact and only categories provided to participants to self-select from, with no breakdown of "Mixed or Other" collected.

© 2025 Braun B et al. *JAMA Netw Open*.

eTable 2. Holm-Bonferroni Multiple Comparisons Adjustment for Trait Anxiety and Depression Models

A

Trait Anxiety

Independent Variable	Dependent Variable	Model *P*	Rank Order of Hypothesis	Holm-Bonferroni adjusted *P*	Hypothesis Testing Decision[a]
Log BMI	STAIC-T	.022	H5	.05	rejected
Log BF%	STAIC-T	.015	H3	.0167	rejected
Log VAT	STAIC-T	.017	H4	.025	rejected
Log lean mass	STAIC-T	.0099	H2	.0125	rejected
VO_2peak	STAIC-T	.005	H1	.01	rejected

B

Depression

Independent Variable	Dependent Variable	Model *P*	Rank Order of Hypothesis	Holm-Bonferroni adjusted *P*	Hypothesis Testing Decision[a]
Log BMI	CDI	.004	H4	.025	rejected
Log BF%	CDI	.005	H5	.05	rejected
Log VAT	CDI	.00004	H1	.01	rejected
Log lean mass	CDI	.002	H2	.0125	rejected
VO_2peak	CDI	.002	H3	.0167	rejected

Abbreviations: BF%, body fat percentage; Lean mass, total body less head adjusted lean mass relative to children's total body less head total body mass; Log; log-transformed; VAT, visceral adipose tissue; VO_2peak, relative VO_2peak oxygen uptake.

[a] Hypotheses are rejected if the model *P* value is less than the Holm-Bonferroni adjusted *P* value.

© 2025 Braun B et al. *JAMA Netw Open*.

eTable 3. Hierarchical Regression Table for Trait Anxiety Outcomes of Complete Data Sample[a]

	Trait Anxiety									
	B	***SE***	***β***	***t***	***95% CI***	***p***	***Adj R²***	***Change in R²***	***F***	***DF***
**Step 1**						0.049[b]	0.020[b]		3.07[b]	(2, 204)
Sex	0.75	0.90	0.06	0.84	(-1.02 to 2.53)	0.40				
Tanner	1.76	0.72	0.17	2.44[b]	(0.34-3.19)	0.02[b]				
**Step 2 a**						0.02[b]	0.032[b]	0.017	3.27[b]	(3, 203)
Log BMI	4.42	2.33	0.14	1.90	(-0.17 to 9.02)	0.06				
**Step 2 b**						0.01[b]	0.036[b]	0.021[b]	3.58[b]	(3, 203)
Log BF%	3.28	1.55	0.15	2.12[b]	(0.23-6.33)	0.04[b]				
**Step 2 c**						0.02[b]	0.035[b]	0.020[b]	3.48[b]	(3, 203)
Log VAT	0.93	0.45	0.15	2.05[b]	(0.04-1.82)	0.04[b]				
**Step 2 d**						0.01[c]	0.040[c]	0.025[b]	3.89[c]	(3, 203)
Log lean mass	-7.99	3.44	-0.16	-2.32[b]	(-14.77 to -1.21)	0.02[b]				
**Step 2 e**						0.005[c]	0.047[c]	0.032[c]	4.41	(3, 203)
VO_2peak	-0.16	0.06	-0.19	-2.63[c]	(-0.28 to -0.04)	0.009[c]				

Abbreviations: BF%, body fat percentage; Lean mass, total body less head adjusted lean mass relative to children's total body less head total body mass; VAT, visceral adipose tissue; VO_2peak, relative VO_2peak oxygen uptake.

[a] Findings are shown for the hierarchical linear regression models with trait anxiety as the outcome measure. Step 1 includes sex as this demographic covariate was included in all models. No other demographic covariates significantly correlated with the mental health outcome (Figure 1). Step 2 include the following variables: (a) log-transformed BMI, (b) log-transformed body fat percentage, (c) log-transformed visceral adipose tissue, (d) log-transformed lean mass, and (e) VO_2peak.

[b] Denotes statistical significance of $P < 0.05$ and 95% CI not including 0.

[c] Denotes statistical significance of $P < 0.01$ and 95% CI not including 0.

© 2025 Braun B et al. *JAMA Netw Open*.

eTable 4. Hierarchical Regression Table for Depression Outcomes of Complete Data Sample[a]

Depression

	B	*SE*	*β*	*t*	*95% CI*	*p*	*Adj R^2*	*Change in R^2*	*F*	*DF*
**Step 1**						0.009[c]	0.036[c]		4.82[c]	(2, 204)
Sex	0.19	0.40	0.03	0.48	(-0.59 to 0.97)	0.63				
Tanner	0.98	0.32	0.22	3.10[c]	(0.36-1.61)	0.002[c]				
**Step 2 a**						0.004[c]	0.049[c]	0.018	4.54[c]	(3, 203)
Log BMI	2	1.02	0.14	1.96	(-0.01 to 4.03)	0.051				
**Step 2 b**						0.005[c]	0.047[c]	0.016	4.4[c]	(3, 203)
Log BF%	1.27	0.68	0.13	1.86	(-0.08 to 2.61)	0.06				
**Step 2 c**						< 0.001[d]	0.093[d]	0.062[d]	8.08[d]	(3, 203)
Log VAT	0.72	0.19	0.27	3.74[d]	(0.34-1.11)	< 0.001[d]				
**Step 2 d**						0.002[c]	0.055[c]	0.023[b]	4.97[c]	(3, 203)
Log lean mass	-3.41	1.51	-0.15	-2.6[b]	(-6.39 to -0.43)	0.02[b]				
**Step 2 e**						0.002[c]	0.054[c]	0.023[b]	4.9[c]	(3, 203)
VO_2peak	-0.06	0.03	-0.16	-2.25[b]	(-0.11 to -0.01)	0.03[b]				

Abbreviations: BF%, body fat percentage; Lean mass, total body less head adjusted lean mass relative to children's total body less head total body mass; VAT, visceral adipose tissue; VO_2peak, relative VO_2peak oxygen uptake.

[a] Findings are shown for the hierarchical linear regression models with depression as the outcome measure. Step 1 includes sex (included in all models) and Tanner's pubertal developmental scale as demographic covariates due to their significant correlations with the outcome mental health measure (Figure 1). Step 2 includes the following variables: (a) log-transformed BMI, (b) log-transformed body fat percentage, (c) log-transformed visceral adipose tissue, (d) log-transformed lean mass, and (e) VO_2peak.

[b] Denotes statistical significance of $P < 0.05$ and 95% CI not including 0.

[c] Denotes statistical significance of $P < 0.01$ and 95% CI not including 0.

[d] Denotes statistical significance of $P < 0.001$ and 95% CI not including 0.

© 2025 Braun B et al. *JAMA Netw Open*.

© 2025 Braun B et al. *JAMA Netw Open*.
