# Supplementary material for: Body Composition, Fitness, and Mental Health in Preadolescent Children
Source: JAMA Netw Open. 2025 Aug 26;8(8):e2528868. doi: 10.1001/jamanetworkopen.2025.28868 (PMC12381672; doi:10.1001/jamanetworkopen.2025.28868)
Supplement: Supplement 2. — Data Sharing Statement [file jamanetwopen-e2528868-s002.pdf]

# Data Sharing Statement

Braun. Body Composition, Fitness, and Mental Health in Preadolescent Children. *JAMA Netw Open*. Published August 26, 2025. doi:10.1001/jamanetworkopen.2025.28868

**Data**
**Data available:** No

**Additional Information**
**Explanation for why data not available:** Data set will be made available from the corresponding author upon reasonable requests, following the publication of the primary outcomes (predicted 2025).
